# Echo Intensity Band Analysis for Assessing Skeletal Muscle Quality in Older Women with Type 2 Diabetes and Peripheral Arterial Disease

**DOI:** 10.3390/jfmk11040377

**Published:** 2026-09-23

**Authors:** Filipi Manoel Silva, Luiz Henrique Rufino Batista, Silas Nery de Oliveira, Francisco Thalyson Moraes Silveira, Fábio Juner Lanferdini, Mateus Rossato

**Affiliations:** 1 Human Performance Laboratory, Faculty of Physical Education and Physiotherapy, Federal University of Amazonas, Manaus 69077-000, Brazil; filipimanoel2013@gmail.com (F.M.S.); henrique.batista@ufam.edu.br (L.H.R.B.); thalyson.moraes22@gmail.com (F.T.M.S.); 2 Biomechanics Laboratory, Center of Physical Education and Sports, Federal University of Santa Maria, Santa Maria Rio Grande do Sul 97105-900, Brazil

**Keywords:** Diabetes Mellitus Type 2, peripheral arterial disease, echo intensity, muscular quality

## Abstract

**Background:** Type 2 Diabetes Mellitus (T2DM) and Peripheral Arterial Disease (PAD) are associated with impaired skeletal muscle quality; however, the impact of T2DM alone and the additional burden of concomitant PAD on echo intensity (EI) band distribution remain unclear. This study evaluated these associations in older women using EI band analysis. **Methods:** Twenty-four older women were allocated into three age-matched groups: GT2DM + PAD (n = 8), GT2DM (n = 8), and healthy participants (HPG; n = 8). Ultrasound images of the vastus lateralis (VL), Tibialis Anterior (TA), Rectus Femoris (RF), and Medial Gastrocnemius (MG) were obtained. Muscle quality was assessed by pixel distribution across EI bands (0 to 255 A.U.) **Results:** In the VL, the GT2DM + PAD showed a significantly lower percentage of pixels in the 0–50 band compared with GT2DM (*p* = 0.020) and HPG (*p* = 0.039), and higher percentages in the 51–100 band (*p* = 0.018 and *p* = 0.030, respectively). For the MG, both GT2DM and GT2DM + PAD exhibited fewer pixels in the 0–50 band and higher values in the 51–100, 101–150, and 151–200 bands than HPG (*p* < 0.05). **Conclusions:** T2DM, alone or combined with PAD, was associated with altered skeletal muscle quality, characterized by a shift toward higher EI bands, suggesting increased non-contractile tissue content.

## 1. Introduction

Interest in the use of echo intensity (EI), assessed through ultrasonography, as an indicator of body composition and muscle quality has increased in recent years among sport and rehabilitation scientists [1]. The fundamental premise for understanding the concept of EI is that muscle tissue is not composed exclusively of contractile proteins, but also contains non-contractile elements such as adipose tissue and fibrous tissue [2]. When analyzed in brightness mode (B-mode), ultrasound images produce a range of pixel intensities, in which skeletal muscle appears in darker shades, whereas adipose and fibrous tissues appear brighter [3]. Traditionally, scales of arbitrary units between 0 (black) and 255 (white) have been used to classify the intensity of the pixels that form the image, where low EI values would indicate greater muscle quality and higher EI values would be an indicator of impairment or disease [4].

Recently, some authors have suggested a new interpretation for EI, which would involve grouping the concentration of pixels by bands [5,6]. According to these authors, the separation by bands could provide additional information on the muscle composition evaluated by ultrasonography. However, a larger body of evidence must be produced to confirm or refute such findings. Current evidence indicates that aging [6] regarding immobilization [5] results in a shift in pixel percentages from the darker (higher quality) bands to the lighter (lower quality) bands. Despite these advances, there are no known studies that have evaluated the percentage distribution of pixels in different EI bands in groups that have chronic diseases alone and in combination, especially Type II Diabetes Mellitus (T2DM) and Peripheral Arterial Disease (PAD).

Although several studies have investigated the EI of patients with T2DM [7,8,9,10], PAD [11], and both [12], to the best of our knowledge, there is no knowledge about the effects of the association of pathologies on the distribution of pixel percentages in the different bands. Our hypothesis is that the association between the pathologies (T2DM + PAD) leads to a reduction in the percentages of pixels in the darker bands (higher muscle quality), redirecting these pixels to lighter bands (lower muscle quality). Our findings may provide new insights into the isolated and associated role of chronic diseases on muscle quality. Therefore, the aim of this study was to evaluate the effect of T2DM alone, as well as the additional impact of concomitant PAD in older women with T2DM, on the percentage pixel distribution across different EI bands.

## 2. Methods

### 2.1. Study Design

This cross-sectional study was disseminated among older women (>60 years) who participated in a physical activity program for the elderly. Interested older women received detailed information about the assessment, and those who volunteered to participate in the study signed a free and informed consent form.

Two hundred and sixty women were selected and intentionally allocated into three groups based on the characteristics of each experimental group. The initial screening involved type 2 diabetes (HbA1c ≥ 6.5%) and Peripheral Arterial Disease (ankle-brachial index < 0.90). Older women with T2DM + PAD was selected to form the first group, followed by older women in the same age group who had only T2DM. Finally, older women with a similar age to the other groups, but without T2DM or PAD, were selected as the healthy participants group (HPG). Among the inclusion criteria, the following were considered: (a) being over 60 years of age; (b) practice the physical activities of the program with a frequency greater than or equal to 3 times a week in the physical activity program for the older. Participants who presented (a) flu-like symptoms, (b) incomplete vaccination schedule against COVID-19, (c) diagnosis of T2DM with open pressure ulcer and/or amputation of lower limbs, (d) peripheral neuropathy, and (e) arterial calcification and incompressible arteries were excluded from the study. The selected older women underwent additional blood tests to confirm the presence or absence of T2DM and determine the lipid profile. Data collection was performed in a single visit to the laboratory, where ultrasound images of the vastus lateralis (VL), Tibialis Anterior (TA), Rectus Femoris (RF), and Medial Gastrocnemius (MG) muscles of the right lower limb were performed. Body fat percentage was determined by air displacement plethysmography. All evaluation procedures were approved by the local Human Research Ethics Committee (CAAE: 51460821.1.0000.5020).

### 2.2. Diagnosis of T2DM and PAD

All participants who met the inclusion criteria underwent laboratory tests to assess hemoglobin A1C (HbA1C) concentrations and lipid profile. HbA1C values ≥ 6.5% or fasting plasma glucose ≥ 126 mg/dL [13] were used as criteria for the diagnosis of T2DM. Ankle-brachial index (ABI) values ≤ 0.90 were considered as criteria for the diagnosis of PAD [14]. The collection procedure was performed with the participant in the supine position, in which she remained at rest for 5 min before the measurement. In the upper limbs, the sphygmomanometer cuff was comfortably positioned above the antecubital fossa, with the cuff directed towards the path of the brachial artery. The cuff was inflated and the maximum pressure was measured by auscultation with a stethoscope. In the lower limbs, the sphygmomanometer cuff was positioned in the middle third of the leg, above the ankles, with the cuff initially facing the posterior tibial artery and, later, towards the pedal artery to measure systolic pressure values. The equipment was inflated until the sound detected by the Doppler became inaudible, and the maximum blood pressure was recorded in the deflation. The procedure was performed twice for each arterial measurement. In case of a difference equal to or greater than 10 mmHg between the upper or lower limbs, another measurement was performed. Subsequently, the ABI was calculated using the formula: higher ankle systolic blood pressure (mmHg)/brachial systolic blood pressure (mmHg). Both lower limbs were evaluated, since the ABI result may be different in each limb. Regarding the scores, values lower than 1 indicate different degrees of arterial insufficiency. Individuals with ABI ≤ 0.90 in both legs were classified as having PAD and were invited to participate in the T2DM + PAD group. Individuals with an ABI of 0.91–1.00 were considered borderline PAD and were excluded. ABI values > 1.00–1.40 were considered normal.

### 2.3. Body Composition Assessment

To determine the percentage of body fat (%F) and the percentage of lean body mass (%FFM), air displacement plethysmography was used with the BOD POD^®^ equipment (Life Measurement Inc., Concord, CA, USA). Before the evaluations, the equipment was calibrated according to the manufacturer’s recommendations. After calibration, the volunteers were evaluated wearing as little clothing as possible and a cap on their heads. To determine body density, the variations between pressure and volume were analyzed by the equipment. Based on this data, the percentages of body fat and lean body mass were calculated using the Siri equation [15].

### 2.4. Muscle Quality Assessment

All older women were instructed not to practice any vigorous physical activity for 48 h before the tests. Before the measurements, the older women rested in the supine position with the lower limbs relaxed and extended for 15 min to allow fluid displacements to stabilize. Ultrasound images were obtained by real-time ultrasound (Mindray DP-30, Global Headquarters, Shenzhen, China). All ultrasonographic measurements were performed with a 38 mm linear transducer with a frequency of 9.0 MHz (depth 40 mm, gain 90 dB). Three cross-sectional images of the VL, RF, TA, and MG muscles were obtained. The analyzed site corresponded to 50% of the thigh length (VL and RF), 30% proximal of the leg length (TA and MG). All images were taken on the right side. A water-soluble gel was used to provide acoustic contact, taking care not to compress the dermal surface. To ensure quantitative image analysis, the Dynamic Range (DR) setting was maintained at a fixed level of 90dB, and non-linear post-processing features (e.g., image processing curves and dynamic contrast enhancements) were disabled. Time gain compensation (TGC) was kept in a neutral position across all depths, and images were exported in uncompressed DICOM format. All system configuration parameters were kept constant (the same) throughout the study and for all participants. All US images were taken by a single experienced evaluator. The images were transferred to a computer for processing. The images were evaluated with the aid of the ImageJ software (version: 1.54t) (NIH—National Institute Health, Bethesda, MD, USA) by a single experienced researcher. The area of analysis was determined by positioning the largest possible rectangle on the image of the evaluated muscle, avoiding adipose tissue and circulating fascia. The histogram produced from the ImageJ software was exported to a Microsoft Excel spreadsheet to express the EI bands at 50 AU intervals (0–50, 51–100, 101–150, 151–200, and 201–255). The number of pixels in each band was then expressed as a percentage of the total pixel content in the histogram. This strategy makes it possible to normalize images that have varying numbers of pixels. The mean values of 3 images was used. This procedure was like that used in other studies [5,6].

### 2.5. Statistical Analysis

All results were presented using descriptive statistics (means and standard deviations). The normality of the data was evaluated by the Shapiro–Wilk test and the homogeneity of the variance by the Levene test. Welch and Brown–Forsythe tests were used to verify the consistency of the data. Subsequently, one-way ANOVAs were carried out to compare the different groups in relation to: (a) characteristics of each group; (b) mean EI in the different groups (HPG, GT2DM and GT2DM + PAD); and (c) percentage of pixels in the different bands (0–50 A.U., 51–100 A.U., 101–150 A.U., 151–200 A.U., 201–255 A.U.). In both, when significant differences were detected, Tukey’s HSD post hoc was applied. The d Cohen and eta squared (η^2^) was used to calculate the effect size. We used the classification criteria proposed by Cohen, where values d = 0.20 or η^2^ = 0.01 are small (S), d = 0.5 or η^2^ = 0.06 are medium (M) and d = 0.80 or η^2^ = 0.14 or more are large (L), respectively. All statistical procedures were performed using the SPSS 27.0 statistical package (IBM, Chicago, IL, USA) for Windows. Because the percentage distribution of pixels across EI bands is compositionally interdependent and involves multiple comparisons across muscles, these analyses were explicitly treated as exploratory. Accordingly, *p*-values are interpreted cautiously alongside effect sizes (η2 and Cohen’s d) to describe patterns of muscle composition shifts.

## 3. Results

The characteristics related to anthropometric, blood, and hemodynamic parameters of the older participants are presented in Table 1. The results indicated significant differences for GT2DM + PAD and GT2DM when compared to HPG in the variables HbA1C (*p* < 0.01), ABI-RP (*p* < 0.01), and ABI-RT (*p* < 0.01). Significant differences were also found between ABI-RT of GT2DM + PAD in relation to HPG (*p* < 0.01) and GT2DM (*p* < 0.01).

The results of the mean EI and the percentage of pixels in the bands for all the muscles of the lower limb analyzed are shown in Figure 1. Regarding the mean EI, significant differences were observed only in the MG [F = 24.533, *p* < 0.001, Eta^2^ = 0.700 (L)], where both the GT2DM (*p* < 0.05) and the GT2DM + PAD (*p* < 0.05) presented values statistically higher than those observed for the HPG (Figure 1G). These results indicate poorer muscle quality in these groups. For the other muscles, no significant differences were observed between the groups evaluated. Regarding the percentage of pixels in each of the bands, significant differences were observed in the 0–50 A.U. band of the VL [F = 5.240, *p* < 0.05, Eta^2^ = 0.333 (L)] where GT2DM + PAD presented statistically lower percentages of pixels when compared to GT2DM (*p* = 0.020) and HPG (*p* = 0.039) (Figure 1B). The opposite was observed in the 51–100 A.U. band [F = 5.570, *p* < 0.05, Eta^2^ = 0.347 (L)], where the GT2DM + PAD presented higher percentages when compared to the T2DM (*p* = 0.018) and HPG (*p* = 0.030). No differences were found between the other bands for the groups evaluated. These results indicate that GT2DM + PAD lost a percentage of pixels in the darker band (higher quality) and gained pixels in the lighter band (lower quality). Significant changes in the MG were also observed (Figure 1H). In the 0–50 A.U. band [F = 7.404, *p* < 0.05, Eta^2^ = 0.414 (L)], both GT2DM (*p* = 0.048) and GT2DM + PAD (*p* = 0.003) presented statistically lower values in the percentage of pixels when compared to HPG. In the band 51–100 A.U. [F = 6.997, *p* < 0.05, Eta^2^ = 0.400(L)], both the GT2DM and GT2DM + PAD groups presented statistically higher values (*p* < 0.05) when compared to the HPG. Similar results were also observed in the bands 101–150 A.U. [F = 6.088, *p* < 0.05, Eta^2^ = 0.367(L)] and 151–200 A.U. [F = 5.099, *p* < 0.05, Eta^2^ = 0.327 (L)], where both GT2DM and GT2DM + PAD presented statistically higher values (*p* < 0.05) when compared to HPG. In summary, the data indicate that the GT2DM and GT2DM + PAD groups lost a percentage of pixels in the darker band (higher quality) and gained pixels in the lighter bands (lower quality).

Table 2 shows the percentage differences and the respective effect size between the different groups, bands and muscles evaluated. In VL, GT2DM + PAD has 12.06% fewer pixels than HPG and 13.46% fewer pixels than GT2DM in the 0–50 AU band. However, in the 51 to 100 AU band, GT2DM + PAD showed opposite behavior, presenting values 12.09% higher than HPG and 13.16% higher than GT2DM. In RF, GT2DM + PAD had 7.64% fewer pixels in the 0–50 A.U. band and a 6.79% increase in the 51–100 A.U. band when compared to GT2DM. In MG the GT2DM has a lower percentage of pixels in the 0–50 A.U. band, when compared to HPG (−8.87%) and GT2DM + PAD (−13.20%). However, GT2DM when compared to HPG has a higher pixel percentage in the bands 51–100 A.U. (8.69%) and 101–150 A.U. (0.18%). GT2DM + PAD when compared to HPG has a higher pixel % band 51–100 A.U. (12.53%), 101–150 A.U. (0.63%) and 151–200 A.U. (0.04%). Finally, GT2DM + PAD, when compared to GT2DM, had a higher percentage of pixels in the bands 151–200 A.U. (0.45%) and 201–255 A.U. (0.04%).

## 4. Discussion

The aim of the present study was to evaluate the association between T2DM, both alone and with concomitant PAD, and the percentage distribution of pixels across different EI bands in older women. Our results partially confirmed our expectations. In the vastus lateralis (VL), although mean EI did not differ significantly between groups, the band analysis revealed that the T2DM + PAD group exhibited a ~13% reduction in pixels in the 0–50 A.U. band (higher quality) in favor of the 51–100 A.U. band (lower quality), compared to both the HPG and T2DM groups. In the medial gastrocnemius (MG), both the T2DM and T2DM + PAD groups displayed higher mean EI compared to the HPG, accompanied by a migration of pixels from the darker, higher-quality bands to the lighter, lower-quality bands.

Traditionally, mean EI has been used as an indicator of muscle quality, where higher values would indicate the presence of non-contractile elements such as adipose tissue and fibrous tissue, indicating lower muscle quality [2]. Studies investigating mean EI in patients with T2DM indicate higher values (lower muscle quality) in RF when compared to healthy older women [8], in addition to an inverse relationship between mean EI and isometric strength of knee extensors [7]. Other studies have sought to investigate mean EI in patients with T2DM who had other associated diseases, such as peripheral neuropathy [9] and observed that RF, AT, and MG had higher mean EI values (lower muscle quality) when compared to the group with only T2DM. These findings are like those observed by Abdulkareem and colleagues [10]. The authors reported that patients who had both T2DM and peripheral neuropathy had higher mean EI values (lower muscle quality) in the TA and abductor hallux when compared to patients who had only T2DM and a control group.

In addition, the authors reported that the isolated presence of T2DM was also associated with higher mean EI values in these muscles compared to the control group. To the best of our knowledge, only Silveira and colleagues [12] assessed mean EI in older women with T2DM alone and in combination with PAD, concluding that the presence of T2DM alone resulted in higher mean EI values (worse muscle quality) compared to the healthy participants group. Despite the use of mean EI as an indicator of muscle quality [6], it is speculated that EI band analysis may better differentiate the specific constituents of muscle tissue. This occurred after the authors observed that the aging process in RF generates a change in the percentage of pixels from the darkest band (0–50 AU) to the lighter bands (51–100 AU). In addition to advancing age, the process of disuse also seems to affect the distribution of pixels in the different bands [5]. The authors observed that 14 days of knee joint immobilization generated a reduction of 3.11% in the 0–50 AU range and increases of 2.94% in the 101–150 AU range and 0.93% in the 151–200 AU range.

Our data are pioneering in showing that the VL of GT2DM + PAD has a lower percentage of pixels in the darker range (0–50 AU), when compared to HPG (−12.06%) and GT2DM (−13.46%). On the other hand, GT2DM + PAD showed a higher percentage of pixels in the lighter range (51–100 A.U.), when compared to HPG (12.09%) and GT2DM (13.16%). However, the most significant changes were observed in the MG, where both the GT2DM and GT2DM + PAD showed a shift to lighter ranges (lower muscle quality) compared to the HPG. The most significant changes occurred in the range of 0 to 50 A.U., where the observed reduction in relation to HPG was 8.87% for GT2DM and 13.20% for GT2DM + PAD, and in the range of 51–100 A.U., where there was an increase in the percentage of pixels of 8.69% for GT2DM and 12.53% for GT2DM + PAD compared to HPG.

The reasons associated with the changes in the distribution of the pixels are not entirely clear, but changes in muscle composition, including fat accumulation (adipose infiltration), increased fibrous tissue, and chronic inflammation, can be pointed to as responsible for the [16] states in which patients with T2DM have a greater amount of intramuscular fat in the distal muscles compared to people without the disease. Similar behavior has also been reported in patients with PAD [17]. This would partially explain the results observed in the MG, where both the GT2DM and the GT2DM + PAD showed the greatest changes. In addition, authors such as Ferreira and colleagues [18] state that patients with PAD have a high number of inflammatory cells in muscle tissue, a fact also observed among patients with T2DM [19]. Previous studies have shown that increases in EI were associated with the presence of inflammatory processes [20].

From a clinical perspective, understanding changes in skeletal muscle properties is important in older adults with T2DM and PAD, as muscle quality impairment is linked to functional limitations and loss of mobility. Although mean EI remains a widely used surrogate marker, analyzing pixel distributions across specific intensity bands may provide complementary insights into structural characteristics that are not fully captured by average echogenicity alone. Identifying these subtle pixel shifts across intensity bands could help clinicians better characterize muscle composition patterns in research and rehabilitation settings, supporting future studies on targeted interventions aimed at preserving functional autonomy.

### Limitations and Strengths

A limitation of this study is the small number of participants in each group, potentially reducing statistical power. Additionally, a major limitation of this study is the absence of a PAD-only control group. Consequently, our design cannot determine the independent association of PAD in the absence of T2DM, nor can it formally evaluate a T2DM-by-PAD statistical interaction. Our findings are restricted to comparing the isolated effect of T2DM against healthy controls and assessing the additive effect of PAD in patients already diagnosed with T2DM. However, our study also has strengths, such as parity in group characteristics (age, body mass, %FB and %LBM), and the fact that this is the first study to evaluate changes in the difference in echo intensity bands in patients with T2DM, both isolated and associated with PAD. Additionally, the analysis of pixel percentage distributions across multiple EI bands within four different muscles in a relatively small cohort involves multiple comparisons and compositionally interdependent data. Consequently, these findings should be considered exploratory and hypothesis-generating, warranting confirmation in larger, prospective cohorts using multivariate compositional data analysis approaches.

## 5. Conclusions

Our findings indicate that T2DM, alone or with concomitant PAD, is associated with altered skeletal muscle echo-intensity distribution in older women, particularly in the vastus lateralis and medial gastrocnemius. EI-band analysis identified differences in pixel distribution that were not consistently reflected by mean EI, suggesting that this approach may provide complementary information on muscle quality. Further studies with larger samples, PAD-only groups, longitudinal designs, and direct measures of tissue composition and functional outcomes are required to establish its diagnostic, prognostic, and clinical utility.

## Figures and Tables

**Figure 1:**
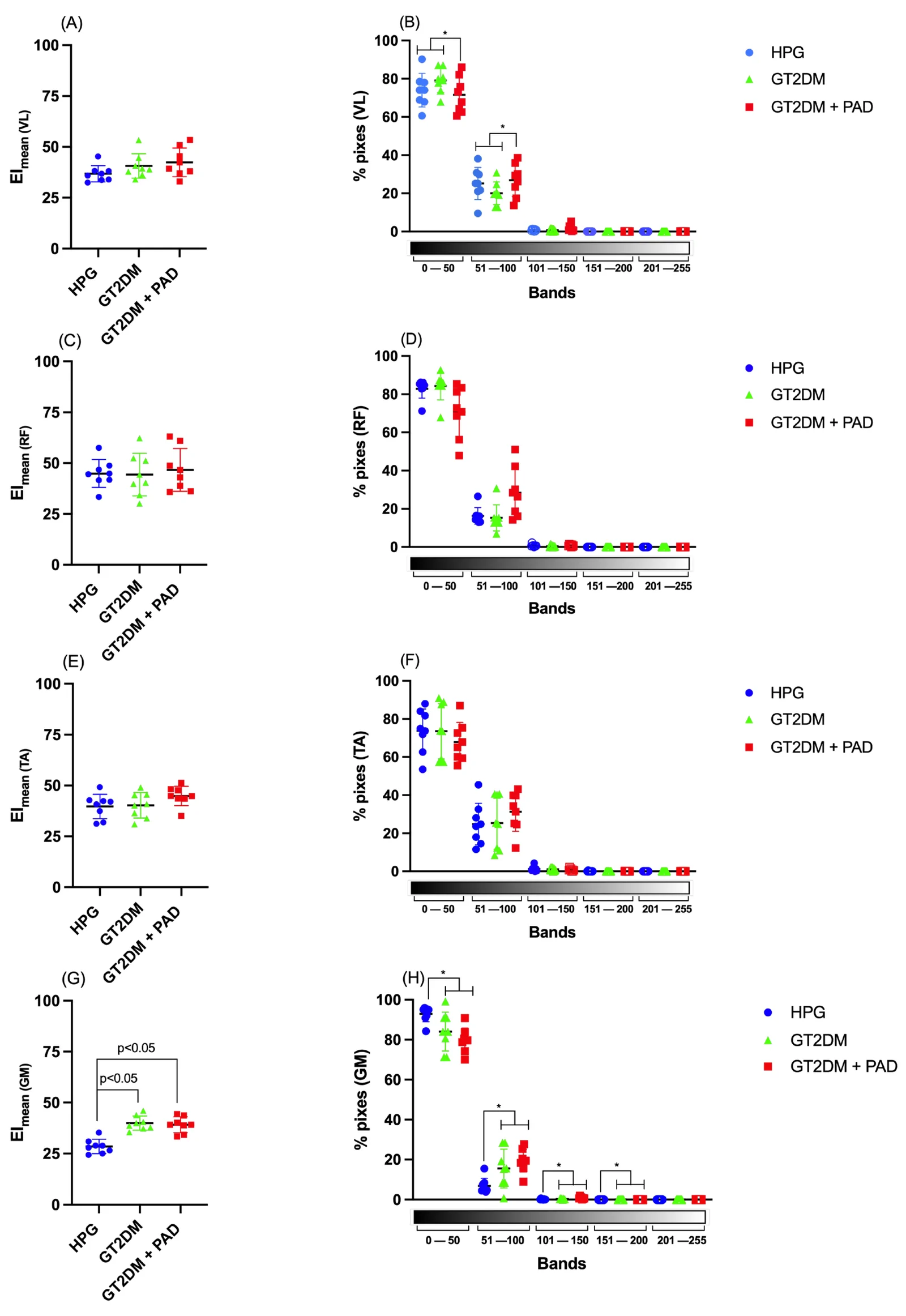
Echo intensity band distribution and mean echo intensity in lower-limb muscles. Mean echo intensity (EImean; left panels) and percentage of pixels distributed across grayscale echo intensity bands (right panels) for the vastus lateralis (VL; (**A**,**B**)), Rectus Femoris (RF; (**C**,**D**)), Tibialis Anterior (TA; (**E**,**F**)), and Medial Gastrocnemius (MG; (**G**,**H**)) in HPG, GT2DM, and GT2DM + PAD. Data are presented as individual values with mean ± SD. * *p* < 0.05 between groups. Abbreviations: EI, echo intensity; VL, vastus lateralis; RF, Rectus Femoris; TA, Tibialis Anterior; MG, Medial Gastrocnemius; A.U., arbitrary units.

**Table 1:** Characterization of participants regarding anthropometric, blood, and cardiovascular parameters.

Variables	GT2DM + PAD (n = 8)	GT2DM (n = 8)	HPG (n = 8)	*p* Value
Age (years)	69.63 ± 8.07	69.38 ± 7.87	69.38 ± 6.65	0.99
Height (cm)	148.12 ± 4.82	151.25 ± 6.45	148 ± 6.23	0.46
Weight (kg)	63.81 ± 8.89	66.33 ± 10.79	58.07 ± 5.29	0.17
%F	40.21 ± 7.05	36.33 ± 6.64	33.76 ± 6.47	0.18
%FFM	59.35 ± 7.44	63.70 ± 6.73	66.23 ± 6.47	0.15
HbA1C(%)	7.66 ± 0.95 *	7.27 ± 1.25 *	5.53 ± 0.48	<0.01
ABI ATA	0.76 ± 0.10 *	1.21 ± 0.10 **	1.08 ± 0.08	<0.01
ABI PTA	0.77 ± 0.11 *	1.11 ± 0.15 **	1.03 ± 0.11	0.001

Notes: %F: Body fat percentage; %FFM: Percentage of fat free mass; HbA1C: Glycated hemoglobin; ABI ATA: Ankle-brachial index—Anterior tibial artery (right); ABI PTA: Ankle-brachial index—Posterior tibial artery (right). * Significant difference compared to the HPG (Post hoc Tukey HSD); ** Significant difference between T2DM and T2DM + PAD group.

**Table 2:** Percentual differences and effect size (ES) between groups (GT2DM + PAD, GT2DM and HPG) in bands (0–50 A.U., 51–100 A.U., 101–150 A.U., 151–200 A.U., 201–255 A.U.) in lower-limb muscles (VL, RF, TA, MG).

Muscle	Groups	0–50 (ES)	51–100 (ES)	101–150 (ES)	151–200 (ES)	201–255(ES)
VL	HPG and GT2DM	+1.40 (0.22-S)	+1.07 (0.18-S)	−0.33 (0.60-M)	−0.01 (0.5-M)	0.00 (0-S)
	HPG and GT2DM + PAD	−12.06 (1.20-L)	+12.09 (1.26-L)	−0.03 (0.05-S)	−0.01 (0.5-M)	0.00 (0-S)
	GT2DM and GT2DM + PAD	−13.46 (1.26-L)	+13.16 (1.28-L)	+0.30 (0.65-M)	0.00 (0-S)	0.00 (0-S)
RF	HPG and GT2DM	+5.14 (0.66-M)	−5.11 (0.70-M)	−0.03 (0.05-S)	0.00 (0-S)	0.00 (0-S)
	HPG and GT2DM + PAD	−2.50 (0.27-S)	+1.68 (0.19-S)	+0.80 (0.63-M)	+0.01 (0.07-S)	0.00 (0-S)
	GT2DM and GT2DM + PAD	−7.64 (0.95-L)	+6.79 (0.92-L)	+0.83 (0.64-M)	+0.01 (0.07-S)	0.00 (0-S)
TA	HPG and GT2DM	−0.27 (0.02-S)	+0.58 (0.04-S)	−0.29 (0.26-S)	−0.02 (0.19-S)	0.00 (0-S)
	HPG and GT2DM + PAD	−5.97 (0.54-M)	+6.48 (0.61-M)	0.51 (0.47-S)	−0.02 (0.19-S)	0.00 (0-S)
	GT2DM and GT2DM + PAD	−5.68 (0.45-S)	+5.90 (0.48-S)	−0.22 (0.30-S)	0.00 (0-S)	0.00 (0-S)
MG	HPG and GT2DM	−8.87 (1.2-L)	+8.69 (1.17-L)	+0.18 (0.81-L)	0.00 (0-S)	0.00 (0-S)
	HPG and GT2DM + PAD	−13.20 (2.56-L)	+12.53 (2.57-L)	+0.63 (1.50-L)	+0.04 (1.13-L)	0.00 (0-S)
	GT2DM and GT2DM + PAD	−4.33 (0.53-M)	+3.84 (0.48-S)	+0.45 (1.03-L)	+0.04 (1.13-L)	0.00 (0-S)

Notes: S—small; M—medium; L—large.

## Data Availability

The datasets generated and/or analyzed during the current study are available from the corresponding author on reasonable request.

## References

[1] Stock M.S., Thompson B.J. (2021). Echo intensity as an indicator of skeletal muscle quality: Applications, methodology, and future directions. Eur. J. Appl. Physiol..

[2] McGregor R.A., Cameron-Smith D., Poppitt S.D. (2014). It is not just muscle mass: A review of muscle quality, composition and metabolism during ageing as determinants of muscle function and mobility in later life. Longev. Heal..

[3] Young H., Jenkins N.T., Zhao Q., Mccully K.K. (2015). Measurement of intramuscular fat by muscle echo intensity. Muscle Nerve.

[4] Manini T.M., Clark B.C., Nalls M.A., Goodpaster B.H., Ploutz-Snyder L.L., Harris T.B. (2007). Reduced physical activity increases intermuscular adipose tissue in healthy young adults. Am. J. Clin. Nutr..

[5] Logeson Z.S., MacLennan R.J., Abad G.-K.B., Methven J.M., Gradl M.R., Pinto M.D., Pinto R.S., Stock M.S. (2022). The impact of skeletal muscle disuse on distinct echo intensity bands: A retrospective analysis. PLoS ONE.

[6] Pinto R.S., Pinto M.D. (2021). Moving forward with the echo intensity mean analysis: Exploring echo intensity bands in different age groups. Exp. Gerontol..

[7] Hirasawa Y., Matsuki R., Tanina H. (2022). Relationship between Echo Intensity of Vastus Lateralis and Knee Extension Strength in Patients with Type 2 Diabetes Mellitus. Phys. Ther. Res..

[8] Nishihara K., Kawai H., Kera T., Hirano H., Fujiwara Y., Ihara K., Kim H., Obuchi S. (2021). Comparisons of muscle thicknesses, echo intensities, and motor functions between community-dwelling older Japanese adults with and without diabetes. Arch. Gerontol. Geriatr..

[9] Zhong Y., Liu X., Lin T. (2023). The Relationship between Muscle Ultrasound Parameters and Diabetic Peripheral Neuropathy among Patients with Type 2 Diabetes Mellitus: A Cross-Sectional Study. J. Diabetes Res..

[10] Abdulkareem S.H., Ameer A.H. (2024). Ultrasonic Evaluation of Muscle Echogenicity in the Lower Limbs for the Detection of Diabetic Peripheral Neuropathy. Front. Biomed. Technol..

[11] Yuguchi S., Ochi Y., Sagata Y., Idesako M., Maeda S., Asahi R., Taniguchi M. (2023). Echo Intensity of Gastrocnemius Is Independently Associated with 6-Minute Walking Distance in Male Patients with Peripheral Arterial Disease. Medicina.

[12] Silveira F.T.M., Ramalho R., Streit I.A., Lanferdini F.J., Rossato M. (2025). Isolated and combined effects of type 2 diabetes mellitus and peripheral arterial disease on muscle architecture, muscle quality, and functional capacity in older women. J. Bodyw. Mov. Ther..

[13] (2014). Standards of Medical Care in Diabetes—2015: Summary of Revisions. Diabetes Care.

[14] Casey S.L., Lanting S.M., Chuter V.H. (2020). The ankle brachial index in people with and without diabetes: Intra-tester reliability. J. Foot Ankle Res..

[15] Siri W.E. (1993). Body composition from fluid spaces and density: Analysis of methods. 1961. Nutrition.

[16] Almurdhi M.M., Reeves N.D., Bowling F.L., Boulton A.J., Jeziorska M., Malik R.A. (2016). Reduced Lower-Limb Muscle Strength and Volume in Patients With Type 2 Diabetes in Relation to Neuropathy, Intramuscular Fat, and Vitamin D Levels. Diabetes Care.

[17] Englund E.K., Langham M.C. (2020). Quantitative and dynamic MRI measures of peripheral vascular function. Front. Physiol..

[18] Ferreira J., Longatto-Filho A., Afonso J., Roque S., Carneiro A.L., Vila I., Silva C., Cunha C., Mesquita A., Cotter J. (2024). Inflammatory Cells in Adipose Tissue and Skeletal Muscle of Patients with Peripheral Arterial Disease or Chronic Venous Disease: A Prospective, Observational, and Histological Study. J. Cardiovasc. Dev. Dis..

[19] Wu H., Ballantyne C.M. (2017). Skeletal muscle inflammation and insulin resistance in obesity. J. Clin. Investig..

[20] Wong V., Spitz R.W., Bell Z.W., Viana R.B., Chatakondi R.N., Abe T., Loenneke J.P. (2020). Exercise induced changes in echo intensity within the muscle: A brief review. J. Ultrasound.

